# Morphological Influence of Solution-Processed Zinc Oxide Films on Electrical Characteristics of Thin-Film Transistors

**DOI:** 10.3390/ma9100851

**Published:** 2016-10-19

**Authors:** Hyeonju Lee, Xue Zhang, Jaeeun Hwang, Jaehoon Park

**Affiliations:** 1 Department of Electronic Engineering, Hallym University, Chuncheon 24252, Korea; zoozs123@naver.com (H.L.); zhangxue00@naver.com (X.Z.); 2 Department of Advanced Materials Engineering for Information & Electronics, Kyung Hee University, Yongin 17104, Korea; jehwang@hanmail.net

**Keywords:** solution process, oxide semiconductor, thin-film transistor, surface morphology

## Abstract

We report on the morphological influence of solution-processed zinc oxide (ZnO) semiconductor films on the electrical characteristics of ZnO thin-film transistors (TFTs). Different film morphologies were produced by controlling the spin-coating condition of a precursor solution, and the ZnO films were analyzed using atomic force microscopy, X-ray diffraction, X-ray photoemission spectroscopy, and Hall measurement. It is shown that ZnO TFTs have a superior performance in terms of the threshold voltage and field-effect mobility, when ZnO crystallites are more densely packed in the film. This is attributed to lower electrical resistivity and higher Hall mobility in a densely packed ZnO film. In the results of consecutive TFT operations, a positive shift in the threshold voltage occurred irrespective of the film morphology, but the morphological influence on the variation in the field-effect mobility was evident. The field-effect mobility in TFTs having a densely packed ZnO film increased continuously during consecutive TFT operations, which is in contrast to the mobility decrease observed in the less packed case. An analysis of the field-effect conductivities ascribes these results to the difference in energetic traps, which originate from structural defects in the ZnO films. Consequently, the morphological influence of solution-processed ZnO films on the TFT performance can be understood through the packing property of ZnO crystallites.

## 1. Introduction

Over the last decade, oxide semiconductors have attracted considerable attention both in academia and industry owing to their high electrical conductivity and excellent optical transparency. It has been reported that the electrical conductivity of these materials can be increased up to 10^3^ S/cm and the optical transparency in the visible-light region is more than 85% [1,2]. These inherent features of oxide semiconductors are highly beneficial for their use in electronics and optoelectronics. In particular, thin-film transistors (TFTs) fabricated with oxide semiconductors hold great promise in a wide range of electronic applications, such as electronic memory devices, sensors, and active-matrix displays [3,4,5]. High-resolution organic light-emitting diode displays that employ an oxide TFT backplane are an example of recent advancement in this technology [6,7]. Now, it is envisioned that solution-processable oxide materials and solution-based manufacturing processes will pave the way for next-generation flexible and disposable electronics.

Among semiconducting oxide materials, zinc oxide (ZnO) is one of the most important binary II–VI compounds because this material can be utilized as the basis of various ternary and quaternary compositions of oxide semiconductors. In addition, ZnO exhibits notable advantages of resource availability, nontoxicity, low cost, and high thermal/chemical stability. It is well recognized that ZnO crystallizes in the hexagonal wurzite structure with preferred *c*-axis orientation; hence, ZnO films are polycrystalline in nature [8]. This is a general observation in solution-processed ZnO films as well as vacuum-processed ones. However, grain boundaries in polycrystalline ZnO thin films can be a main constraint on TFT performance because they act as trapping centers for the mobile charge; the grain boundary corresponds to the area where crystallite grains meet.

In the literature, several scattering mechanisms have been proposed to understand the charge-transport behavior in polycrystalline films: ionized impurity scattering, neutral impurity scattering, grain-boundary scattering, lattice vibration scattering, and intragrain cluster scattering [9,10,11,12,13]. It is found that the ionized scattering and the intragrain cluster scattering are notable in heavily doped semiconductors (a carrier concentration >10^20^ cm^−3^), and the lattice vibration scattering is dominant in the high temperature range. The grain-boundary scattering mechanism is known to be applicable when the mean-free path of the carriers in the films is comparable to the grain sizes. However, neutral impurity scattering is plausible for undoped oxide semiconductors if oxygen vacancies provide the source of neutral impurity scattering [14,15]. Considering that ZnO is a pure/undoped oxide semiconductor, the grain-boundary scattering may be one of the crucial factors that affects the charge transport in polycrystalline ZnO films, especially at room temperature.

Moreover, research efforts have also been devoted to developing a potential barrier model at grain boundaries [11,16,17,18]. This model proposes that electrons are trapped at grain boundaries so that a space-charge region is created in the grains. The resulting negative potential barrier at the grain boundaries thus impedes the motion of electrons. In certain cases, physical device simulations were performed to study the correlation between the location/orientation of grain boundaries and the potential barrier height [19,20]. To date, within the framework of these mechanisms, the doping effects of oxide semiconductors have been properly analyzed and the electrical properties of TFTs with various oxide compositions have been widely characterized. Nevertheless, a physical picture of the morphological influence of solution-processed ZnO films on the TFT performance must still be obtained in terms of structural defects, such as pinholes and voids.

Here, we would like to point out that the solidification of solution-processed films is essentially distinguished from the growth kinetics of vacuum-deposited films. During vacuum deposition processes, nucleation of films occurs from nuclei on the surface of the substrate, thereby allowing the bottom-up growth of films. In the case of solution processes, solutes can be accommodated in the bulk and near the surface of a coated film, while substrate heating causes the solidification to progress from the bottom. This complexity eventually dictates the morphological characteristics of solution-processed films.

In this paper, we report the morphological influence of solution-processed ZnO semiconductor films on the electrical characteristics of ZnO TFTs. Note that the study of solution-processed ZnO semiconductors is of prime importance because ZnO can be the basis in solution-processable oxide semiconductors with various compositions for TFT applications. Different film morphologies were produced by controlling the spin-coating condition of a precursor solution. The TFT performance was explained based on the Hall-measurement results of ZnO films. The operational stability of ZnO TFTs was examined through consecutive measurements. The gate-voltage dependence of the electron transport behavior in the TFT channel was investigated by analyzing the field-effect conductivities. From an energy band model at the dielectric/semiconductor interface, we can explain the morphological influence of solution-processed ZnO films in terms of fixed states in shallow and deep energy levels.

## 2. Results and Discussion

**Figure 1:**
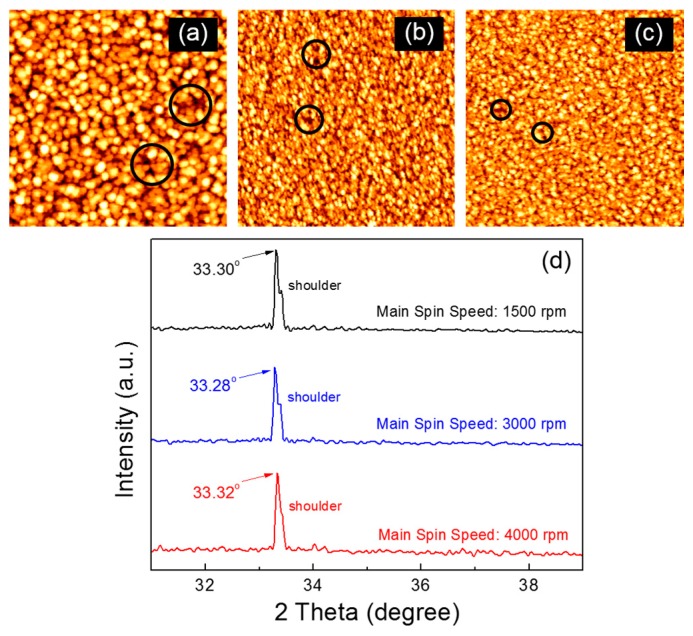
Atomic force microscopy (AFM) images (1 μm × 1 μm) of the ZnO films coated at the main spin speed of (**a**) 1500; (**b**) 3000; (**c**) 4000 rpm. (**d**) X-ray diffraction (XRD) patterns of the fabricated ZnO films.

Figure 1a–c show the atomic force microscopy (AFM) images of the ZnO films that were fabricated through a spin-coating process of the ZnO precursor solution at different main spin speeds. The ZnO film coated at the main spin speed of 1500 rpm exhibits the largest ZnO crystallites, whereas there is no notable difference in the size of ZnO crystallites between those films coated at main spin speeds of 3000 and 4000 rpm. However, the structural defects in the fabricated ZnO films, such as voids and pinholes, appear to differ depending on the coating condition. In our results, such defects were fewer and smaller when the ZnO film was coated at a higher main spin speed. Some of structural defects are indicated by black circles in Figure 1a–c, of which diameters were estimated to be 60 ± 15 nm, 45 ± 10 nm, and 30 ± 8 nm, respectively. In addition, a smoother surface is observed for the ZnO film coated at a relatively higher spin speed. The root mean square roughness values for the ZnO films coated at main spin speeds of 1500, 3000, and 4000 rpm are measured to be 0.76, 0.63, and 0.45 nm, respectively.

It is well recognized that the main spin step during the spin-coating process serves to thin the fluid to form a film, after spreading the dispensed fluid over the substrate. This implies that crystallite coarsening and condensation properties are inevitably affected by the main spin condition. Crystallite coarsening is considered to be favorable at a lower main spin speed, thereby resulting in larger ZnO crystallites and rough film surface as shown in Figure 1a. On the other hand, the smaller ZnO crystallites and a smooth film surface shown in Figure 1b,c suggest that a faster coating of the ZnO solution contributes to the film condensation rather than crystallite coarsening. In other words, smaller ZnO crystallites are formed and their distribution becomes more concentrated in the film when the precursor solution is spin-coated at a relatively higher speed. The different surface morphologies are thus ascribed to the effect of the spin-coating condition on the crystallite coarsening and condensation processes.

Figure 1d compares the X-ray diffraction (XRD) patterns of the fabricated ZnO films. All the ZnO films exhibit the strongest peak at a diffraction angle of approximately 33.3°, corresponding to the (002) plane of ZnO semiconductors with the wurzite structure [21]. This result suggests that the ZnO films were preferentially grown in the *c*-axis direction. The shoulder peak at approximately 33.4° is possibly due to structural defects in the film because it becomes weak with increasing main spin speed. From the XRD spectrum, the average size (*D*) of ZnO crystallites is calculated using Scherrer’s equation:

(1) $$D=\frac{k\lambda }{\beta cos\theta }$$

where *k* is a constant assumed to be 0.94, *λ* is the wavelength of the incident X-rays, *β* is the full width half maximum, and *θ* is the diffraction angle [22]. The calculated crystallite sizes in the ZnO films coated at main spin speeds of 1500, 3000, and 4000 rpm are approximately 99.1, 87.6, and 81.9 nm, respectively. The variation in the XRD-derived crystallite sizes with the coating condition is in agreement with that observed in the AFM images.

**Figure 2:**
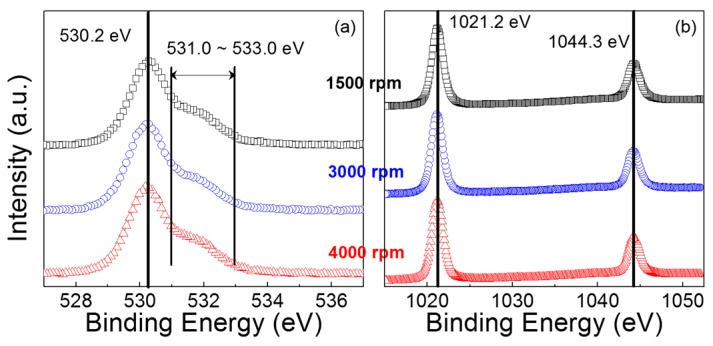
(**a**) O1*s* and (**b**) Zn 2*p* X-ray photoemission spectroscopy (XPS) spectra of the fabricated ZnO films.

X-ray photoemission spectroscopy (XPS) analysis of the ZnO films was performed to investigate their chemical characteristics. Figure 2a shows that the O1*s* XPS spectra are similar for all the films, in which the most intensive peak is positioned at a binding energy of approximately 530.2 eV. This intensive peak at 530.2 eV represents the Zn–O bond that contributes to the concentration of the ZnO lattice due to full oxidation without oxygen vacancies [23]. However, the shoulder in the spectral region between 531.0 and 533.0 eV could have resulted from oxygen deficiency and hydroxide; the characteristic peaks at the binding energies of approximately 531.0 and 532.0 in the O1*s* XPS spectrum are known to originate from the oxygen vacancy and the chemisorbed/dissociated oxygen or OH species, respectively [24,25]. Table 1 summarizes the results of deconvoluting the obtained O1*s* spectra into three components; *O*_I_, *O*_II_, *O*_III_, and *O*_tot_ denote the areas of the components centered at approximately 530.0, 531.0, and 532.0 eV, and the total O1*s* peak area, respectively. The fitting result exhibits no meaningful difference in the peak distribution feature. Notably, the area ratios of *O*_II_/*O*_tot_ are quite comparable for all the films, which indicates that the concentration of oxygen vacancies was not affected by the coating condition in this study. In Figure 2b, each XPS spectrum in the range of the Zn 2*p* orbital peak shows two distinct peaks at binding energies of 1021.2 and 1044.3 eV, corresponding to the binding energies of the 2*p*_3/2_ and 2*p*_1/2_ states, respectively. The spin-orbit splitting energy of 23.1 eV, which is derived from the difference between these two binding energies, suggests that the Zn atoms exist as Zn^2+^ oxidation states in the ZnO film [26,27]. Note that the chemical environment surrounding the Zn atoms is similar for our ZnO films. Additionally, the atomic concentration ratio of O to Zn (i.e., O/Zn) is calculated to be 0.85, 0.83, and 0.84 for the ZnO films coated at main spin speeds of 1500, 3000, and 4000 rpm, respectively. The oxygen contents in the ZnO films are thus expected to be comparable to each other. These results confirm that the chemical characteristics of the fabricated ZnO films were not significantly affected by the coating condition. Accordingly, the performance of the ZnO TFTs can be studied on the basis of the difference in the morphological characteristics of the ZnO films.

**Figure 3:**
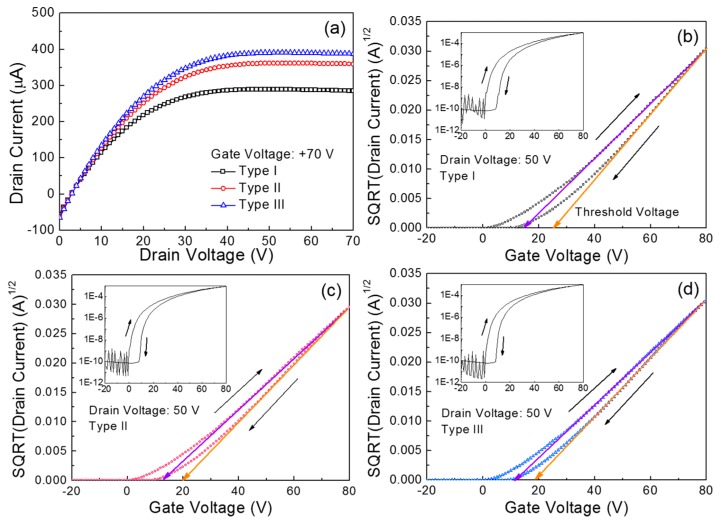
(**a**) Output and (**b**–**d**) transfer characteristics of the fabricated ZnO thin-film transistors (TFTs). The insets show the plots of log |drain current| versus gate voltage.

Figure 3a compares the output characteristics of the fabricated ZnO TFTs. Herein, the three types of TFTs are denoted according to the main spin speed: Type I for 1500 rpm, Type II for 3000 rpm, and Type III for 4000 rpm. These output characteristics were measured under vacuum conditions (~8 mTorr) by changing the drain voltage (*V*_D_) from 0 to 70 V in increments of 1 V at a constant gate voltage (*V*_G_) of 70 V. All the TFTs exhibit a clear pinch-off and an excellent saturation with an *n*-channel enhancement mode operation. These behaviors point to the transportation of electrons in the ZnO semiconductor layer, which is essentially controlled by positive gate and drain voltages. Meanwhile, it is observed that the drain current (*I*_D_) of the Type I TFT is substantially lower than those for the Type II and Type III devices. The drain current values extracted at a drain voltage of 70 V are 284.8 μA for Type I, 359.2 μA for Type II, and 387.5 μA for Type III. Figure 3b–d show the transfer characteristics of the three types of ZnO TFTs. These characteristics were measured under vacuum conditions (~8 mTorr) at a constant drain voltage of 50 V while the gate voltage was swept reversibly from −20 to 80 V in increments of 1 V. An important observation is the hysteretic behavior in the transfer characteristics upon reversing the gate voltage sweep direction, resulting in a positive shift in the threshold voltage (*V*_T_); the threshold voltage was extracted from the corresponding plot of the square root (SQRT) drain current versus gate voltage, as the gate voltage at which the drain current was extrapolated to zero. In particular, the clockwise hysteresis observed for all the TFTs can be ascribed to a charge-trapping phenomenon in the conducting channel of the transistor [28]. The trapped charge concentration (*Q*_t_) in the conducting channel is calculated using the shift in the threshold voltage (Δ*V*_T_) with the following equation:

(2) $$Q_{t}=\Delta V_{T}\times C_{\mathrm{ox}}$$

where *C*_ox_ is the capacitance of the gate dielectric layer [29].

The calculated trapped charge concentrations are summarized in Table 2. Obviously, the largest shift in the threshold voltage for the Type I TFT is relevant to the largest trapped charge concentration. Meanwhile, we observed that such a hysteretic behavior of ZnO TFTs became more severe when the solution-processed ZnO films were produced at higher annealing temperatures (>160 °C). This might be indicative of thermally induced cracks in the solution-processed ZnO films. Thus, the thermal stability issue in solution-processed ZnO TFTs should be addressed in further studies. Besides, the field-effect mobility (*μ*_eff_) in the saturation region is estimated using the following relationship:

(3) $$\mu_{\mathrm{eff}}=\frac{2L\times I_{D}}{nWC_{\mathrm{ox}}}\times \frac{1}{{\left(V_{G}-V_{T}\right)}^{2}}$$

where *nW* is the effective channel width of the TFTs having an interdigitated geometry for the source and drain electrodes, *L* is the channel length, and *C*_ox_ is the capacitance of the gate dielectric layer [29]. The subthreshold swing (SS) was defined as the change in the gate voltage required to change the drain current by a factor of 10. The obtained TFT parameters are also summarized in Table 2. It is clear that the Type III TFT exhibited superior performance, which is consistent with the results of the output characteristics shown in Figure 3a.

**Figure 4:**
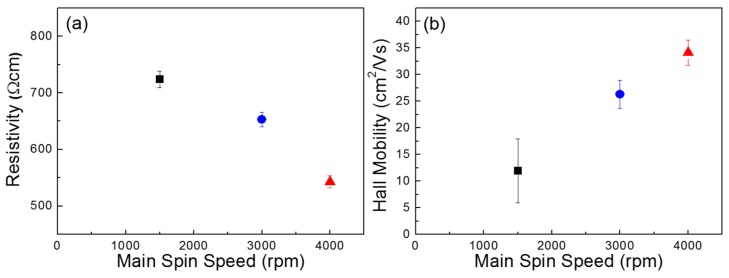
(**a**) Resistivities and (**b**) Hall mobilities of the ZnO films coated at different main spin speeds.

Figure 4 compares the electrical properties of the three sets of the ZnO films, which were obtained by Hall-effect measurement. A lower resistivity and a higher Hall mobility (*μ*_H_) are observed for the film coated at a faster main spin speed. These results demonstrate that the electrical conductance of the ZnO film can be enhanced when the film is coated at a higher main spin speed in our experiments. The enhancement of the electrical properties of the ZnO semiconductor films thus contributes to the TFT performance. Further, the electron concentrations (*N*) of the ZnO films coated at main spin speeds of 1500, 3000, and 4000 rpm were measured to be 3.2 × 10^14^, 8.5 × 10^14^, and 2.7 × 10^15^ cm^−3^, respectively. It should be noted that the variation in the electron concentrations observed in the Hall-effect measurements does not agree with the comparable area ratios of *O*_II_/*O*_tot_ obtained by XPS analysis. This disagreement between the Hall measurement and the XPS analysis results signifies that the film morphology dominantly affects the electrical properties of the film. To attest the charge transport behavior in the ZnO films, the mean free path (*l*) of the carriers is theoretically estimated using the following equation:

(4) $$l=\frac{h}{2e}{\left(\frac{3N}{\pi }\right)}^{1/3}\mu_{H}$$

where *h* is Planck’s constant and *e* is the electron charge [10,30]. Previously, it was reported that the grain-boundary scattering is a dominant mechanism for the carrier mobility when the grain size is comparable with the mean free path [10,13,30,31]. However, the calculated mean free paths for the present ZnO films were less than 1 nm, which is much smaller than the crystallite size observed in Figure 1. This implies that the grain-boundary scattering has little effect on the charge transport behavior in our ZnO films. Hence, energetic traps distributed in the polycrystalline ZnO films can be suggested as a limiting factor for the charge transport in the film. It should also be noted that structural defects (i.e., pinholes and voids) were fewer and smaller when the ZnO film was coated at a higher main spin speed, as shown in Figure 1a–c. Then, we can infer that such structural defects within the ZnO semiconductor create energetic states that can trap charge carriers so that the packing density of ZnO crystallite in the film dictates the electrical properties of the ZnO films and the TFT performance as well.

**Figure 5:**
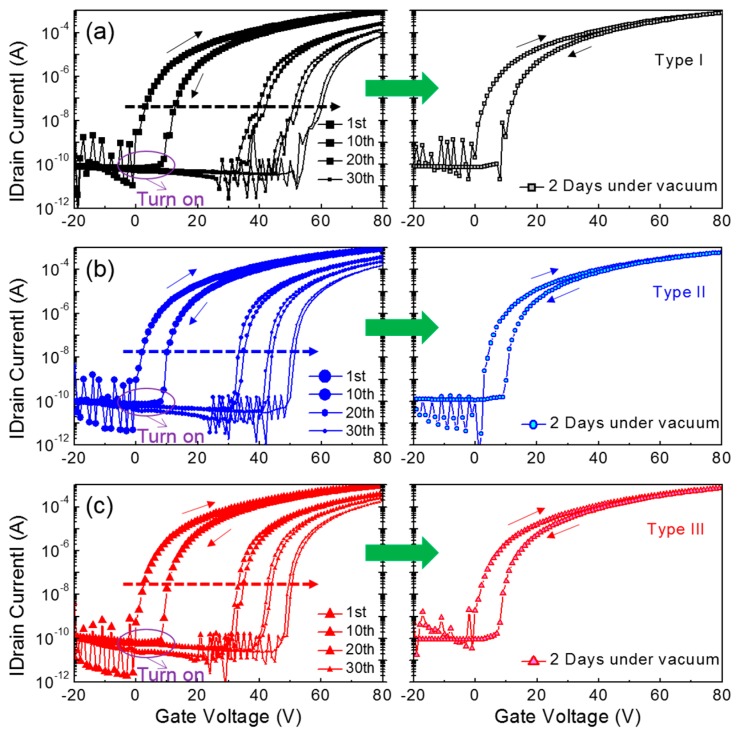
Transfer characteristics obtained during consecutive TFT operations and after storage under vacuum. (**a**) Type I; (**b**) Type II; (**c**) Type III TFTs.

In this study, we also observed the characteristic variations in the fabricated transistors, which occurred during consecutive TFT operations under vacuum conditions (~8 mTorr). In addition to a long-term bias stress at a fixed gate voltage, a short-term bias stress during consecutive TFT operations is important to assess the electrical stability of the TFTs, which determines the optimum driving voltage and switching speed in integrated circuits [32]. Figure 5 shows the transfer characteristics of the three types of ZnO TFTs, which were measured at a constant drain voltage of 50 V while the gate voltage was swept reversibly from −20 V to 80 V in increments of 1 V. After completing the consecutive operations, the TFTs were kept intact under vacuum condition for 2 days and their transfer characteristics were then measured again for the characteristic recovery test. During 30 measurement cycles, the turn-on point of each device was shifted in the positive direction and the hysteretic behavior in the transfer characteristics became vague. However, the TFT characteristics nearly recovered toward the initial value after the devices were stored for 2 days under vacuum conditions (~8 mTorr). Apparently, these changes in the transfer characteristics seemed to be alike for the three types of ZnO TFTs.

**Figure 6:**
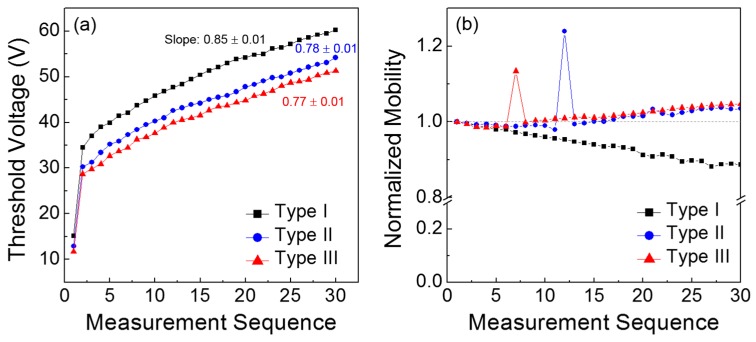
Variations in (**a**) the threshold voltage and (**b**) the field-effect mobility of the ZnO TFTs during consecutive TFT operations.

For a more comprehensive understanding of the results in Figure 5, the obtained TFT parameters, such as threshold voltage and field-effect mobility, are compared as a function of the measurement sequence. From Figure 6a, the positive shift in the threshold voltage is clearly observed for all the TFTs, which is analogous to the positive shift in the turn-on point. In particular, it should be noted that the Type I TFT exhibited the steepest slope among the three types of transistors; the slope was extracted by linear fitting from the 2nd to the 30th values. Because the slope corresponds to the variation in the threshold voltage according to the measurement sequence, the channel formation in the Type I TFT is considered to seriously deteriorate during repetitive operations. Figure 6b presents the change in the field-effect mobility according to the measurement sequence; the field-effect mobility values were extracted in the saturation region of the TFT characteristics, and those values were normalized with respect to the initial value of each case. It is observed that the field-effect mobility of the Type I TFT decreased with repeated measurements, whereas those of the Type II and III devices slightly increased. These different behaviors in the field-effect mobility case should be distinguished from monotonic positive shifts in the threshold voltage shown in Figure 6a. This may manifest the influence of gate bias on the variations in field-effect mobility and threshold voltage during consecutive TFT operations. Noteworthy is that the threshold voltage is extrapolated in the subthreshold regime and the field-effect mobility is extracted above the threshold regime.

In order to investigate the gate-voltage dependence of the charge-transport behavior in the TFT channel, the field-effect conductivity (*σ*) was calculated at a drain voltage of 5 V by using the following equation [33]:

(5) $$\sigma \left(V_{G}\right)=\frac{L}{nW}\times \frac{I_{D}}{V_{D}}$$

**Figure 7:**
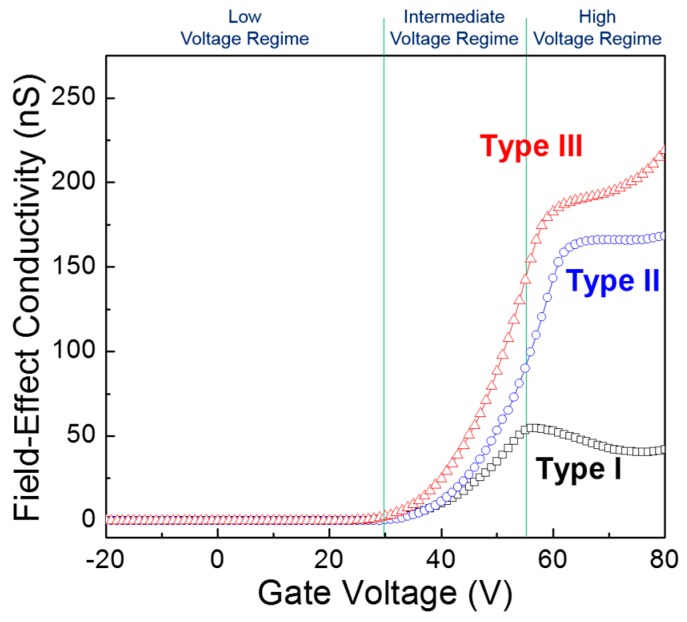
Field-effect conductivities of the three types of ZnO TFTs as a function of gate voltage.

Figure 7 shows the field-effect conductivities as a function of gate voltage for the three types of ZnO TFTs. In the low gate voltage regime (*V*_G_ < 30 V), the field-effect conductivities of the three types of TFTs were not only as low as 0.1 nS but also independent of the gate voltage. In the intermediate gate voltage region (30 V < *V*_G_ < 55 V), the field-effect conductivities substantially increased with increasing gate voltage. These results can be explained by a trap-filling phenomenon which is commonly observed in polycrystalline semiconductor devices [34]. In other words, as more field-induced charges are accumulated in the TFT channel with increasing gate voltage, the energetic barriers among individual ZnO crystallites will decrease by filling energetic traps with field-induced charges, thereby increasing the field-effect conductivities. A higher rate increase in the field-effect conductivity with the gate voltage can thus be ascribed to lower and fewer energetic traps in the ZnO film. In contrast, a more complicated picture is observed in the high gate voltage regime (*V*_G_ > 55 V); a decrease in the field-effect conductivity in the Type I TFT, a rather uniform value in the Type II TFT, and an increase in the field-effect conductivity in the Type III TFT. Here, we hypothesize that charge-trapping sites are produced owing to structural defects, such as pinholes and voids, in the solution-processed ZnO films and thus their energetic depth is ineluctably determined by the packing density of the ZnO crystallites.

**Figure 8:**
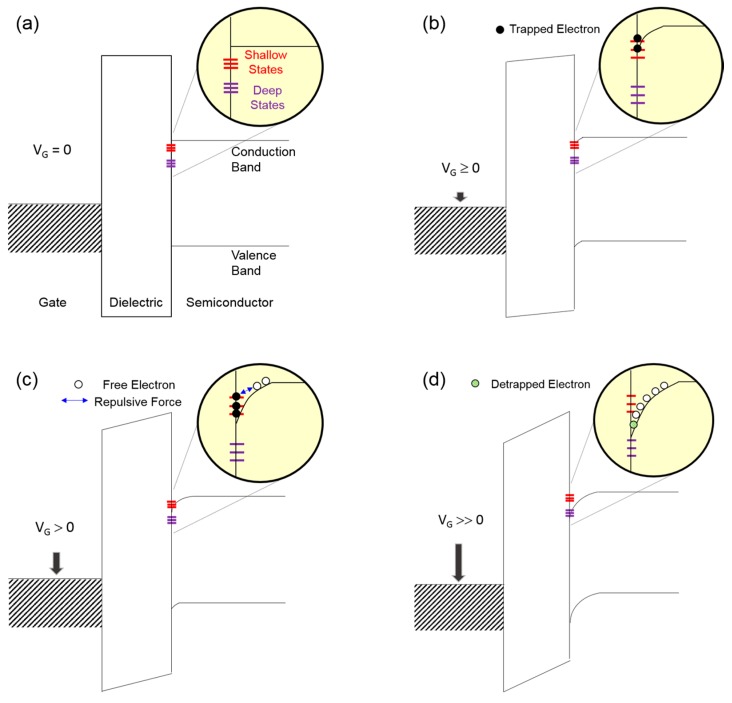
Energy band diagrams of the *p*-doped Si gate/SiO_2_ dielectric/ZnO semiconductor structure according to gate-biasing conditions; (**a**) *V*_G_ = 0 V; (**b**) *V*_G_ ≥ 0 V; (**c**) *V*_G_ > 0 V; (**d**) *V*_G_ >> 0 V.

Figure 8 shows the energy band diagrams of the *p*-doped Si gate/SiO_2_ dielectric/ZnO semiconductor structure according to gate-biasing conditions, in which fixed states with different energetic depth are introduced at the interface between the SiO_2_ dielectric and ZnO semiconductor. In this conceptual approach, we assume that the energy bands are flat under equilibrium conditions, as shown in Figure 8a; the work function difference between the gate metal and the semiconductor is disregarded for simplicity. When a positive gate voltage is applied, the energy bands in both the dielectric and semiconductor should be bent downward, and the resulting positive slope of the semiconductor contributes to the accumulation of free electrons in the vicinity of the SiO_2_ dielectric/ZnO semiconductor interface. As a result of this band bending, interfacial states are possibly filled with free electrons (i.e., negatively charged), suggesting that interfacial states can act as charge-trapping sites. In addition, negatively charged fixed states are expected to produce a negative potential, which is effective at decreasing the applied positive gate voltage. The band bending proceeds with the application of the gate voltage; its gate-voltage dependence is further described in Figure 8b–d. If a low positive gate voltage is applied (*V*_G_ ≥ 0, Figure 8b), a weak band bending leads to filling shallow-level interfacial states with free electrons so that current conduction at the SiO_2_ dielectric/ZnO semiconductor interface is negligible. Figure 8c depicts that shallow interfacial states are fully filled with free electrons because a moderate band bending occurs with increasing gate voltage (*V*_G_ > 0). Excess electrons, which are not trapped at shallow-level states, may flow at the SiO_2_ dielectric/ZnO semiconductor interface. In the meantime, trapped electrons seem to remain nonconductive owing to low kinetic energy. Note that the kinetic energy of an electron existing above the conduction band is defined by the difference between its energy level and the conduction band minimum. Such trapped electrons preferentially interfere with the accumulation of free electrons at the interface by inducing a negative potential. Under a moderate band-bending condition, a higher density of shallow interfacial states may produce a larger negative potential, thereby allowing a lower current flow. In the case of a strong band bending (*V*_G_ >> 0, Figure 8d), trapped electrons now obtain sufficient kinetic energy to escape from shallow interfacial states. Without the influence of deep-level interfacial states, detrapped electrons, together with free electrons, contribute to the current. On the contrary, a net current would be limited again by the existence of deep interfacial states when a strong band bending facilitates the filling of deep-level states with electrons. Finally, the variations in the field-effect conductivities of the three types of ZnO TFTs shown in Figure 7 can be understood through the combined effects of shallow and deep interfacial states. Using this physical model, we found that negatively charged interfacial states inevitably deteriorate the *n*-channel TFT operation because their negative potential interferes with the accumulation and conduction behaviors of electrons at the dielectric/semiconductor interface. It is thus reasonable to state that the positive shift in the threshold voltage shown in Figure 6a is caused by the negatively charged shallow interfacial states. In addition, the decrease in the field-effect mobility for the Type I TFT shown in Figure 6b can be ascribed to the detrimental effect of negatively charged deep interfacial states. These results prove that charge-trapping sites at the SiO_2_ dielectric/ZnO semiconductor interface are created owing to structural defects, such as pinholes and voids, in the solution-processed ZnO film, and thus their energetic depth is dominantly determined by the packing density of ZnO crystallites. From the characteristic recovery tests in Figure 5, it is found that trapped electrons in the vicinity of the TFT channel region can be redistributed in equilibrium during storage without applying a gate bias. Consequently, the comparative analysis of the morphological properties of solution-processed ZnO film and the electrical characteristics of ZnO TFTs suggests that the packing density of ZnO crystallites in the solution-processed film is of prime importance for the performance and reliable operation of TFTs.

## 3. Materials and Methods

**Figure 9:**
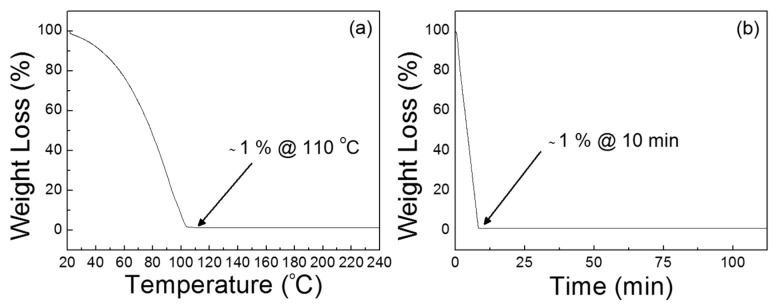
Thermogravimetric analysis (TGA) characteristic curves of the prepared ZnO precursor solution; plots of weight loss versus (**a**) temperature and (**b**) time.

The precursor solution for ZnO semiconductor films was prepared in ambient air by dissolving ZnO powders (Sigma Aldrich, 99.99%, Yongin, Korea) in aqueous ammonia-hydroxide solution (Duksan, 25%–30%, Yongin, Korea). The percentage of ZnO powders in ammonia water was approximately 0.9 wt %. To ensure the precursor solution is stable, the solution was placed in a refrigerator for 12 h after stirring using a magnetic bar for 1 h. Thermogravimetric analysis (TGA) of the precursor solution was carried out using a thermal analysis instrument at a scanning rate of 10 °C/min under a nitrogen ambient. Figure 9a shows that the dehydration of the precursor solution proceeds below 100 °C and the decomposition temperature is approximately 110 °C. The result of isothermal sintering of the precursor solution in Figure 9b also indicates that the thermal annealing of the precursor solution at 110 °C requires more than 10 min in order to produce a ZnO film. The remaining weight percentage was approximately 1% after the isothermal test for 10 min, and the weight loss then occurred at a very slow rate because of the residual hydroxyl groups in the material. From the results, a thermal annealing process at 110 °C for 30 min was adopted to produce ZnO films from the present precursor solution.

**Figure 10:**
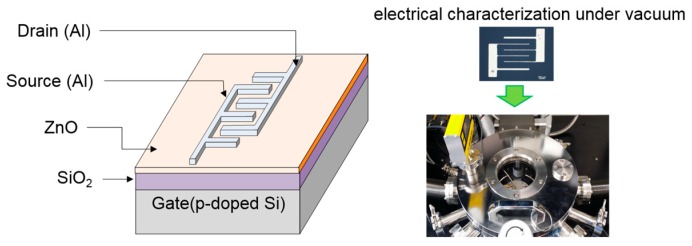
Schematic representation of the fabricated ZnO TFT. Optical microscope image and picture display the fabricated TFT and the vacuum probe station system, respectively.

Solution-processed ZnO TFTs were fabricated on a *p*-doped silicon substrate with a 100-nm-thick silicon dioxide (SiO_2_) dielectric layer. Figure 10 shows the bottom-gate/top-contact structure of TFTs in which source and drain electrodes were formed with an interdigitated geometry. Prior to TFT fabrication, the substrate was cleaned in an ultrasonic bath using acetone, isopropyl alcohol, and deionized water in sequence for 30 min each. Then, an oxygen plasma treatment was performed on the substrate for 10 s to produce a hydrophilic surface which is essential for interface suitability between the SiO_2_ dielectric and ZnO semiconductor layers; for the oxygen plasma treatment, a radio-frequency power of 50 W was applied with an oxygen flow rate of 10 sccm. The precursor solution was filtered through a 0.45 μm syringe filter and was spin-coated on the substrate. In our study, the main spin speed during the spin-coating process was varied among 1500, 3000, and 4000 rpm to produce different morphologies of ZnO films. Our spin-coating process consisted of the three steps: the first dispensing step at a spin speed of 500 rpm for 5 s, the second coating step at each main speed for 30 s, and the final drying step at a spin speed of 500 rpm for 5 s; a ramp time of 1 s was applied for the acceleration and deceleration processes. The ZnO semiconductor film was fabricated after thermal annealing of the coated film at 110 °C for 30 min in air. The resulting thicknesses of the ZnO films coated at main spin speeds of 1500, 3000, and 4000 rpm were approximately 24, 20, and 18 nm, respectively. Finally, 50 nm thick Al source and drain electrodes with an interdigitated geometry were thermally deposited through a shadow mask under a base pressure of 6 × 10^−6^ Torr. The interdigitated electrodes consisted of five finger pairs with 125 μm channel spacing and an electrode width of 440 μm. Thus, the effective channel length and width in the fabricated TFTs were 125 μm and 2200 μm, respectively.

TGA (N-1000, SCINCO, Seoul, Korea) measurement was performed to monitor the chemical reaction in the ZnO precursor solution. XPS (K-Alpha, Thermo Scientific, Waltham, MA, USA) measurement was performed to observe the chemical properties of the ZnO films. The morphological and structural characteristics of the ZnO films were investigated using AFM (XE150, PSIA, Santa Clara, CA, USA) and XRD (DMAX-2500, Rigaku, Tokyo, Japan) measurements. Hall measurements using the van der Pauw configuration were used to measure the electrical resistivity and electron concentration of the ZnO films. Electrical characterization of the ZnO TFTs was carried out in the dark and under vacuum using a semiconductor parameter analyzer (4200-SCS, Keithley, Seoul, Korea).

## 4. Conclusions

We investigated the morphological influence of solution-processed ZnO semiconductor films on the electrical characteristics of ZnO TFTs. It was shown that a densely packed ZnO semiconductor film with reduced structural defects, such as pinholes and voids, contributed to the TFT performance by providing a low electrical resistivity and a high Hall mobility. When these transistors were subjected to consecutive TFT operations, the positive shift in the threshold voltage was invariably observed for all the TFTs, but the variation in the field-effect mobility seemed to correlate with the morphological properties of the solution-processed ZnO semiconductor films. These different behaviors between the threshold voltage and the field-effect mobility were thought to have originated from the fact that the threshold voltage is in the subthreshold regimes and the field-effect mobility is extracted above the threshold regime. Importantly, the results of the field-effect conductivities in the ZnO TFTs indicated that electron transport in the TFT channel is essentially determined by the gate voltage and is ineluctably affected by the morphological properties of the ZnO semiconductor film. From the experimental results, we could demonstrate an energy band model that explains the gate-voltage dependence of the accumulation and conduction behaviors of electrons at the SiO_2_ dielectric/ZnO semiconductor interface. Consequently, it is concluded that structural defects in the solution-processed ZnO film create fixed states with various energetic depths at the dielectric/semiconductor interface, and negatively charged interfacial states deteriorate the *n*-channel operation of ZnO TFTs. This underlines the significance of the packing density of ZnO crystallites in the solution-processed film. To reach a more definite understanding of the impact of defect-created interfacial states on the performance of oxide TFTs, it is necessary to quantitatively analyze both state distributions and concentrations in the energy level. We believe that these results can be a basis for further studies on the stability and reliability of solution-processed oxide TFTs.

## Figures and Tables

**Table 1:** Deconvolution results of O1*s* XPS spectra for the fabricated ZnO films.

Main Spin Speed	(*O*_I_ Region) Binding Energy *O*_I_*/O*_tot_ %	(*O*_II_ Region) Binding Energy *O*_II_*/O*_tot_ %	(*O*_III_ Region) Binding Energy *O*_III_*/O*_tot_ %
1500 rpm	530.2 ± 0.02 eV 69.9%	530.9 ± 0.02 eV 16.2%	532.1 ± 0.02 eV 13.9%
3000 rpm	530.2 ± 0.02 eV 69.4%	531.0 ± 0.01 eV 16.1%	532.0 ± 0.02 eV 14.5%
4000 rpm	530.2 ± 0.02 eV 69.2%	531.2 ± 0.03 eV 16.5%	532.2 ± 0.02 eV 14.3%

**Table 2:** Performance parameters of the three types of ZnO TFTs.

TFT	*Q*_it_	*V*_T_	*μ*_eff_	SS
Type I	2.4 × 10^12^ cm^−2^	14.2 V	0.68 cm^2^/Vs	1.6 V/decade
Type II	1.6 × 10^12^ cm^−2^	12.6 V	0.72 cm^2^/Vs	1.4 V/decade
Type III	1.2 × 10^12^ cm^−2^	11.5 V	0.80 cm^2^/Vs	1.3 V/decade
